# Spoof surface plasmon based planar antennas for the realization of Terahertz hotspots

**DOI:** 10.1038/srep18606

**Published:** 2015-12-22

**Authors:** Yusheng Zhang, Zhanghua Han

**Affiliations:** 1 Centre for Terahertz Research, China Jiliang University, Hangzhou 310018, China

## Abstract

Novel spoof surface plasmon based terahertz (THz) antennas are realized using a few number of rectangular grooves perforated in ultrathin metal stripes and the properties of them, including both scattering cross sections and field enhancement, are numerically analyzed. The dependence of these properties on the incident angle and groove number is discussed and the results show that sharp resonances in scattering cross section spectra associated with strong local field enhancement can be achieved. These resonances are due to the formation of Fabry-Perot resonances of the spoof surface plasmon mode and it is found that the order of resonance exhibiting strongest field enhancements is found to coincide with the number of grooves at normal incidence, due to hybridization of the antenna resonance with the individual groove resonance. The terahertz hotspots within the grooves at resonances due to the local field enhancement may open up new possibilities for the investigation of terahertz-matter interactions and boost a variety of THz applications including novel sensing and THz detections. The planar stripe antennas with sharper resonances than dipolar-like resonances, together with their ease of fabrication may also promise new design methodology for metamaterials.

For decades, the so-called ‘terahertz gap’ between 0.1 THz and 10 THz have been less explored than those neighboring bands in the electromagnetic wave spectrum mainly because of the lack of efficient sources and detectors working in this regime, which thus hinders the rapid technological progress in this field. Nevertheless, due to the unique features of THz waves such as low photon energy and nonionizing radiation, numerous applications have been widely investigated including non-destructive biosensing, medical imaging and space communication, etc^1^,^2^,^3^. More importantly, many chemicals and molecules have their vibrational and rotational resonance frequencies located in the THz regime, resulting in unique absorption spectral features, which can be used for fingerprint detection of specific chemicals. These applications, especially when a broad spectral band is required for chemicals whose fingerprint resonances extend over several terahertzes, mainly rely on the use of THz time-domain spectroscopy (THz-TDS)^4^,^5^. In the THz-TDS systems the THz radiations are usually generated by use of a femtosecond laser pulse incident onto a photoconductive antenna or an electro-optic rectification crystal to generate the THz pulses. However, due to the low efficiency of the optical-THz conversion in photoconductive antennas or the low efficiency in the nonlinear optical processes involved in the optical generation of THz pulses, the resulted THz signal is usually not strong enough in terms of spectral power density over a large bandwidth and the spectral information obtained with THz-TDS can be easily blurred by the environmental turbulence. As a result, the widespread use of THz-TDS in the applications of fingerprint detections is heavily hindered and some critical conditions like cryogenic temperatures are sometimes required to achieve an acceptable single to noise ratio.

These limitations, however, can be circumvented by enhancing the interactions between molecules and THz radiations by increasing the local electromagnetic field so that even when the THz signal is not very strong, a significant spectral feature of the molecular absorption can still be expected. One of the effective and practical ways of realizing local field enhancement is to use metallic structures whose dielectric functions are negative to support the excitation of surface plasmon (SP) modes^6^. The coupling of electromagnetic (EM) fields with coherent oscillations of metal’s free electrons will exhibit strong field confinement on the subwavelength scale. Two famous examples of using this mechanism have been successfully demonstrated include surface enhanced Raman scattering (SERS) at optical frequencies^7^ and surface-enhanced infrared absorption (SEIRA) in the infrared^8^. In the THz and microwave frequencies, because the metal permittivity approaches negative infinity and EM waves cannot be bound to the metal surfaces, regular SP modes are not supported using smooth metals and the properties of regular SPs cannot be directly transferred. Many material platforms, including highly doped semiconductors^9^,^10^ and graphenes^11^, have been exploited and investigated for the realization of SP modes among which the corrugated metal surfaces which support the artificial surface waves termed as “spoof (or designer) surface plasmons (SSP)”^12^,^13^, are of special interest due to the ease of use for practical applications compared to other candidates^14^,^15^.

Although the concept of SSP has been proposed for almost one decade, its use in the form of localized SSP to enhance the local field has attracted more research interest only since recently. Actually, localized SSP supported by resonant antenna structures are of special interest for THz sensing applications owing to the large field enhancement occurring at the resonances. In the past few years, periodically textured closed surfaces^16^ and cavities^17^ have been proposed and experimentally demonstrated at microwave frequencies^18^ to support localized SSPs, which resemble the localized surface plasmons (LSPs) in the optical frequencies. Meanwhile, multi-band localized SSPs and magnetic localized surface plasmons have also been numerically investigated by decorating periodically textured cylinder and closed textured cavities^19^,^20^,^21^. However, most of the above mentioned structures are quite complicated in shape. Even though they can be manufactured using mechanical drilling at large size for microwave applications, it will be very difficult using mechanical method to fabricate those curved structures working in terahertz regime in which the wavelength is shorter, thus limiting the fabrication of three-dimensional structures on metal surfaces and the use of them in practical THz photonic applications.

Quite recently, we proposed a two-dimensional (2D) concept of retardation-based corrugated metal stripe antennas and numerically demonstrate that sharp spoof surface plasmon resonances with strongly local field enhancement can be achieved in the THz regime^22^. In this paper we extend this idea of SSP based stripe antennas to the three dimensional (3D) case using a few rectangular grooves perforated through metal stripes with very small thickness and analyze the scattering properties. This kind of thin structured metal stripe has been investigated to support the conformal SPs for waveguiding over long distances in the THz and microwave frequencies^23^ and has the main advantage of easy manufacturing using regular fabrication techniques over those investigated e.g. in refs 13,14,16,17. By terminating the groove array perforated in the metal stripes to keep only a few grooves, the SSP based stripe antennas can be realized. Some spectral characteristics of these antennas including the scattering cross section and the field enhancement due to the excitation of the SSP are numerically investigated. The dependence of these characteristics on groove number and incidence angle of excitation THz waves show that the sharpest resonance happens when the order of resonance coincides with the groove number, probably due to a hybridization of the SSP mode between the transverse electromagnetic (TEM) modes propagating within individual grooves. The sharp resonances with these antennas compared to those regular dipole resonances may open up new possibilities for metamaterial design. Surprisingly, the strongest field enhancement is achieved using a small groove number of three at normal incidence, showing that an array composed of these antennas seems very promising for sensing applications in the terahertz or microwave frequencies.

The formation of the antennas is due to the excitation of SSP mode propagating along the corrugated metal stripes and getting reflected at both terminations to form the Fabry-Perot (FP) modes. So in this section, we first analyze the dispersion property of one-dimensional (1D) rectangular groove array perforated in an ultrathin metal film. The unit cell of the groove array is composed of a rectangular groove with a fixed width *W* = 0.5 *P*, opening to bottom distance *H* = *P* perforated through a thin metal stripe with thickness assumed to be *t*. The bottom of the groove is separated from the edge of the metal stripe with a distance *h* = 0.2 *P*, as can be seen in the schematic in Fig. 1(a), so that only a single SPP mode is present along the groove array. In this paper P is chosen as 50 *μm* so that all the resulted characteristic frequencies lie in the THz regime, although the frequencies in all the figures below are still shown normalized to *c*/*P* where *c* is the light speed in vacuum. All the spectra in this paper are within the range of 0.02 *c*/*P* ~ 0.15 *c*/*P*, which corresponds to a frequency domain between 0.12 THz and 0.9 THz. In our simulations, the surrounding background medium is assumed to be homogeneously polymide with the permittivity as 2.9. To facilitate the investigated structure in practical use, the metal stripe thickness *t* is set as 2 *μm* which is easy to realize using metal evaporation in fabrication. This value is nearly zero-thickness compared to the wavelengths up to a few hundred microns in THz. The dispersion relation for the SSP mode supported by this periodic groovy array is calculated using a finite-element method (FEM) based eigen-frequency solver and the result is shown as the dashed line in Fig. 1(b), where the straight solid line is given for the light in the background medium for comparison. In the calculation of the dispersion, the metal is assumed to be perfect electric conductor (PEC) for simplicity. From Fig. 1(b) one can see that the ultrathin corrugated metal stripe exhibits a well-pronounced SP-like behavior. The dispersion curve moves to the right when the thickness decreases, showing a trend of tighter modal confinement.

When the corrugated metal stripe perforated with groove array is truncated to be consisting of a few grooves, as is shown schematically in Fig. 1(a), the SSP mode will experience reflections at both ends and propagate back and forth along the corrugated metal stripe to form the FP modes. In this case the truncated waveguiding configuration will exhibit some antenna behavior and resonates only at certain frequencies. If the groove number in the antenna is *N*, the total length *L* of the antenna should be *L* = *(N* + *0.5)* *P taking into account of the metallic walls at both ends with width of 0.5 *P* to form the metallic closure. Intuitively, the resonating condition will be^24^,


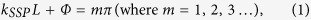


where *k*_*SSP*_ is the wavevector of the SSP mode, *L* is the length of stripe antenna, *m* is a positive integer indicating the resonance order, and Φ is the reflection phase at both terminations due to the partial scattering of the SSP modes into free space. When this condition is fulfilled, resonating characteristics including enhanced scattering and field enhancement will be envisioned. To validate our prediction, a planar antenna composed of three grooves with the parameters specified above is investigated numerically using 3D finite element method (FEM) which is implemented in the commercial software of Comsol Multiphysics. In the simulations, to make the results more guiding for real applications, the metal is assumed to be copper with a conductivity of 5.8 × 10^7^ S/m. The scattering field from this antenna illuminated by a plane wave incident normal to the antenna with a linear polarization along the antenna (*x* direction) is first studied. The calculated scattering cross sections (SCSs) normalized to the surface size of the antenna perpendicular to the incidence direction are given in Fig. 1(c) as a function of frequency, which is given with the unit of f_0_ = *c*/*P*. It is clearly observed that there are two SCS resonance peaks in the investigated spectral range, the first one at 0.0510 f_0_ while the other one at 0.1075 f_0_. From the dispersion curve we can find that wavevector of the SSP mode *k*_*SSP*_ is 0.11 * (2π/P) at the frequency of 0.051 f_0_ while for the third resonance order *k*_*SSP*_ is 0.38 * (2π/P) at the frequency of 0.1075 f_0_. One can calculate that with these values the left side of equation (1) is roughly equal to the right side with some minor discrepancy due to partial reflections at both ends indicating that there are related to a Fabry-Perot mode. From the mode profiles at these two frequencies (the electric field distribution E_x_ at 0.1075 f_0_ is given in Fig. 1(d)), one can judge that these two frequencies correspond to two FP modes with the mode order m equaling to 1 and 3 respectively. At the frequency of 0.0510 f_0_ Fig. 1(b) indicates that the wavevector of SSP mode *k*_*spp*_ does not deviate from the wavenumber in polymide so remarkably, so the antenna working at this frequency is quite like a regular half-wavelength dipole antenna, with a broad SCS resonance and moderate field enhancement. The mode profile at this resonance also resembles a dipole antenna very much. What is more interesting is the second resonance at 0.1075 f_0_, which shows a SCS resonance although weaker but much sharper compared to the first one. The highest electric field amplitude enhancement across the antenna structure at this resonance is above 250 times higher than the incident plane wave, showing that a significantly enhanced electric field has been achieved in this antenna structure. Note that the electric field intensity which is critical for many applications will be enhanced by a factor of 62500.

It is worth noting that the field enhancement, as can be seen in Fig. 1(c), is much stronger at the FP resonance order of 3 than that at lower order. Interestingly, this order number coincides with the groove number of the antenna. To get more insight into the details, we plot in Fig. 1(d) the electric field distribution (E_x_) of second resonance at the central *xy* plane through the metal. Besides the pattern of *m* = 3 FP resonance at the lower metal edge, one can also see that the transverse electromagnetic (TEM) mode supported by individual groove are also strongly excited in three grooves. Actually one can see strong hotpots of electric field close to the bottom of each groove (in the *xy* plane), which represent the highest field enhancement. It is also seen that the center of the three grooves are very close laterally to the center of the three antinodes in the FP resonance, indicating that these two modes might be correlated. Judging from the mode distribution and the fact that the highest field enhancement happens at the FP resonance number coinciding with the groove number (this can also be verified for antennas composed of other groove numbers, which will be shown later), one can conclude that the strong field enhancement is due to the hybridization of the antenna resonance with the individual groove resonance.

One can find in Fig. 1 that only the FP resonances with odd order numbers of 1 and 3 are excited while mode with an even order number *m* = 2 is absent. This is due to symmetry reasons. When the plane wave is incident normal to the antenna top surface, the overlap between the plane wave and the even order FP resonance mode which has an asymmetrical profile will be zero, thus this resonance cannot be excited. We further investigate the cases when the incident wave is oblique. The *k* vector of the plane wave is fixed within the *xz* plane but has an angle of *θ* with respect to the normal of the antenna surface. In Fig. 2(a), the normalized SCS spectra of metal stripe antenna composed of three grooves are presented for the incident angle of θ equaling to 0, 30, 45 and 60 degrees. The SCS is normalized by the effective surface size normal to the incidence, i.e. *(H* + *h)* * *Lcosθ*. One can see that for the oblique incidence, one additional resonance appears 0.0890 f_0_ between the two original resonances shown above. The mode profile and the result from equation (1) both confirm that this additional resonance is due to the excitation of FP mode with order *m* = 2. When comparing the even resonance with odd resonance, one can see that even mode presents a symmetric Lorentz-like resonance line shape, while the odd modes behave as asymmetric Fano resonance, quite similar to case of the multiple order FP resonances supported by long metallic nanorods, which was experimentally observed in optical frequencies^25^,^26^. The results in Fig. 2(a) also show that for odd order of resonances the excitation efficiency will decrease as the incident angle increases while for the even number of FP resonances the highest SCS appears at the angle of 45 degrees at which the overlap between the incident wave and the FP mode reaches its highest value.

Figure 2(b) compares the electric field enhancement at different frequencies for both the normal incidence and a single oblique incident angle of 30°. One can see that an oblique incidence, although can excite the even order of FP modes, degrades the electric field enhancement for the odd number of modes significantly, especially for *m* = 3, at which this antenna composed of *N* = 3 grooves has its highest field enhancement. So one can conclude that for antennas composed of odd number of grooves, to get the highest enhancement it is advantageous to use normal incidence. For antennas with even number of grooves, an oblique incidence is required to excite the mode first.

The same idea of constructing SSP based novel antennas can be extended to other groove numbers. In order not to lose generality, we will show the cases in the following part the groove number N equaling to an even number of 4 and odd number of 5 respectively. For a higher groove number, one can expect from equation (1) that more FP mode orders will appear as an increase of the antenna length. Figure 3(a) presents the calculated normalized SCS and field enhancement as a function of frequency for N = 4 at normal incidence. Apparently only the FP modes with odd orders of 1 and 3 are excited in this case, and the frequencies of these two orders have a red-shift due to an increase of the total antenna length. The top figure on the right panel of Fig. 3 shows the distribution of H_z_ for the FP resonance order of *m* = 3. It is seen that three antinodes appear at the lower edge of metal cross section while there is a mismatch between the antinode number and groove number in the upper region. As a result, the highest electric field intensity enhancement drops to around 48400, a bit lower than that can be reached with antenna composed of three grooves. For *N* = 5, the results in Fig. 3(b) show that only FP resonance with orders of *m* = 1, 3, 5 are excited, with an increasing electric field intensity enhancement as FP order number increases and reaches around 32400 when the order number equaling to the groove number. The magnetic field H_z_ distributions at the last two orders are shown in the middle and lower figures on the right panel of Fig. 3, which clearly show the order number.

Similar to the even order of FP resonance shown in Fig. 2, the *m* = 2 and 4 orders of FP resonances can be excited by using oblique incidence for these two antennas. Our simulation results reveal that for the antenna composed of 4 grooves, when the incident angle is 45° which is expected to excite the *m* = 4 order of FP resonance most efficiently, the field enhancement at this resonance is only a factor of around 30625, still less than that for the order of *m* = 3 at normal incidence. This is probably because at oblique incident only part of the plane wave is effective in the coupling with the FP mode. This result implies that in order to get the highest, antennas composed of odd number of grooves working at normal incidence is preferred.

One may also notice that for antennas with groove number of 3, 4, 5 at normal incidence, the highest electric field enhancement decreases with the groove number. This means that to have a higher field enhancement at normal incidence it is advantageous to use antennas composed of less grooves. This is useful for real applications in which an array of such antennas is normally used. A smaller unit cell of such an array may be of special interest for normal incidence because there are no higher order modes of diffraction and the incident THz radiation can be made the best use of.

For real applications periodic antenna arrays are usually used to facilitate the excitation and measurement of the sample. The inset in Fig. 4 shows the unit cell of such a two-dimensional array consisting of N = 3 antennas fabricated on the polymide substrate and covered by the same material as well. The period along the *x* and *y* direction are chosen as 4 P (200 μm) and 1.6 P (80 μm) respectively so that one can get a near-zero transmission for the resonances of our interest. A normal THz incident along the *x* direction is used and the calculated transmission spectrum is presented as the red line in Fig. 4. The first resonance located at around 0.07 c/P in this antenna array is blue shifted from the original resonance 0.0510 f_0_ shown in Fig. 1(c) due to the near-field coupling between adjacent periods. The second resonance remains almost the same position as shown in Fig. 1(c) because this FP resonance is highly localized within individual grooves and near-field coupling between periods can be minimized. A third resonance which is not shown in Fig. 1 now appears in the periodic structure and this resonance shifts remarkably compared to the other two resonances as the period along *x* direction changes. This resonance is due to diffraction resulting from the scattering by the periodic arrangement of these antennas, which has been found as the collective resonance in nanoparticle arrays in optics^27^. These finds further confirm that the resonances presented in Figs 1, 2, 3 are FP modes due to the SSP propagating along the antennas.

In conclusion, we have theoretically demonstrated that retardation-based planar stripe antennas on an ultrathin corrugated metallic stripe with a few grooves can support multi-order localized spoof surface plasmon resonances at terahertz frequencies. Simulation results clearly show the sharp Fabry-Pérot resonances in scattering cross section spectra together with strongly local field enhancement. These antennas have planar structures with the metal thickness of only 2 *μm*, thus are quite easy to fabricate using regular fabrication techniques like photolithography and metal deposition, showing great advantages over those literally proposed THz disk or cylindrical counterparts. What’s more, the highest electric field intensity using these antennas has been enhanced with respect to the incident wave by an order of four even taking into account of real metal losses. These results suggest that the SSP based THz antennas can work as a good supplement to THz grapheme antennas^28^ to provide an excellent platform for the investigation of THz nonlinear optics and light-molecular interactions^29^ in the THz regime and boost a variety of THz applications including novel sensing with high Q factor^30^,^31^,^32^ and THz detections. The sharper SCS resonances at higher order FP resonances compared to dipolar-like resonances supported by regular metal stripes may also provide a new method for metamaterial design.

## Additional Information

**How to cite this article**: Zhang, Y. and Han, Z. Spoof surface plasmon based planar antennas for the realization of Terahertz hotspots. *Sci. Rep.* **5**, 18606; doi: 10.1038/srep18606 (2015).

## Figures and Tables

**Figure 1:**
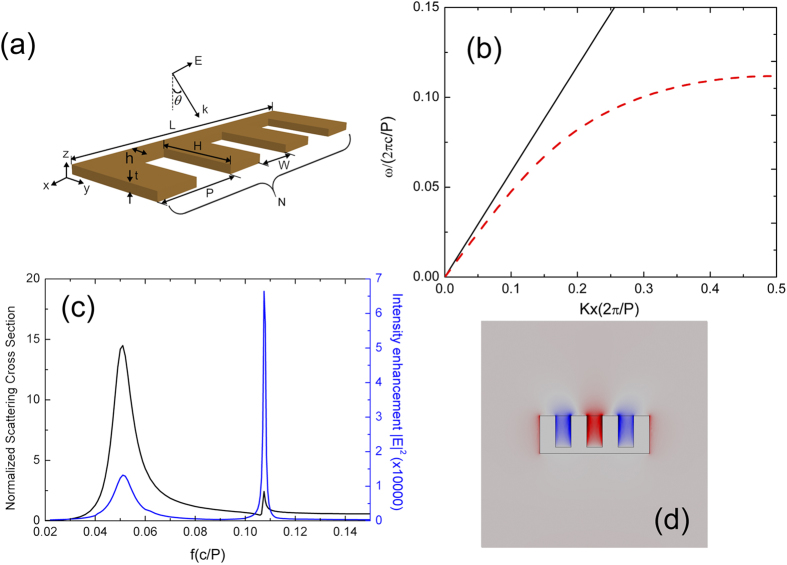
(**a**) Schematic of the three-dimensional corrugated metal stripe antenna structure (**b**) Calculated dispersion relation for the SSP mode supported by an infinite number of grooves for the metal stripe thickness *t* = 2 μm. (**c**) Normalized scattering cross section and field enhancement as a function of frequency. (**d**) The distribution of electric field (Ex) at the central *xy* plane of the metal stripe.

**Figure 2:**
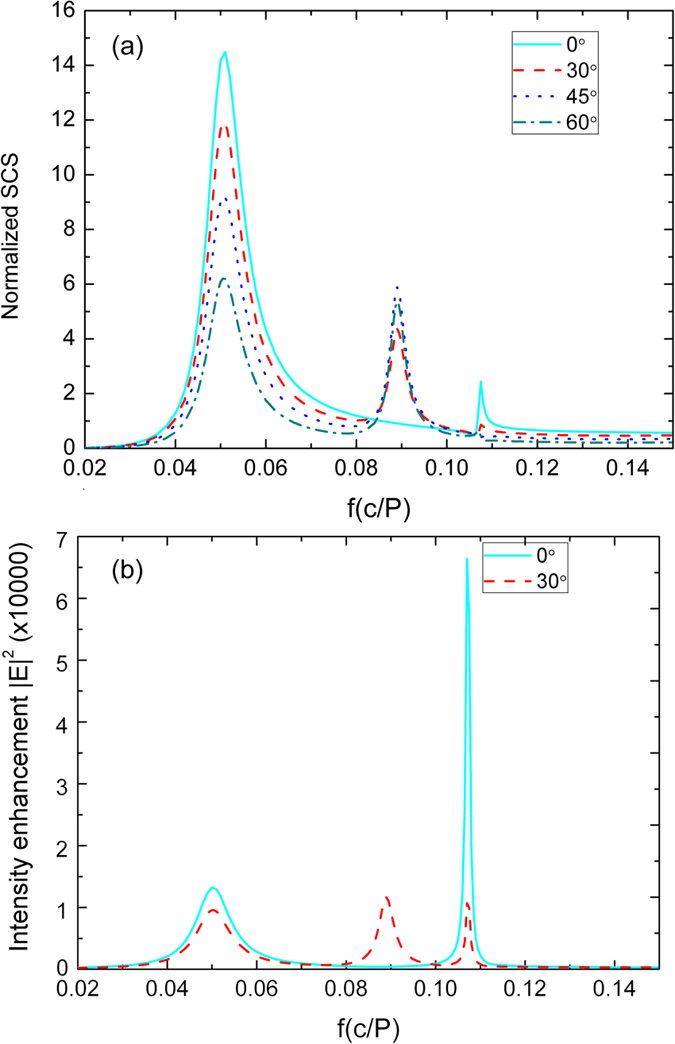
(**a**) Normalized SCS spectra of metal stripe antenna with *N* = 3 for varying angle of incidence θ. (**b**) The electric field enhancement for different incidence angle 0° and 30°.

**Figure 3:**
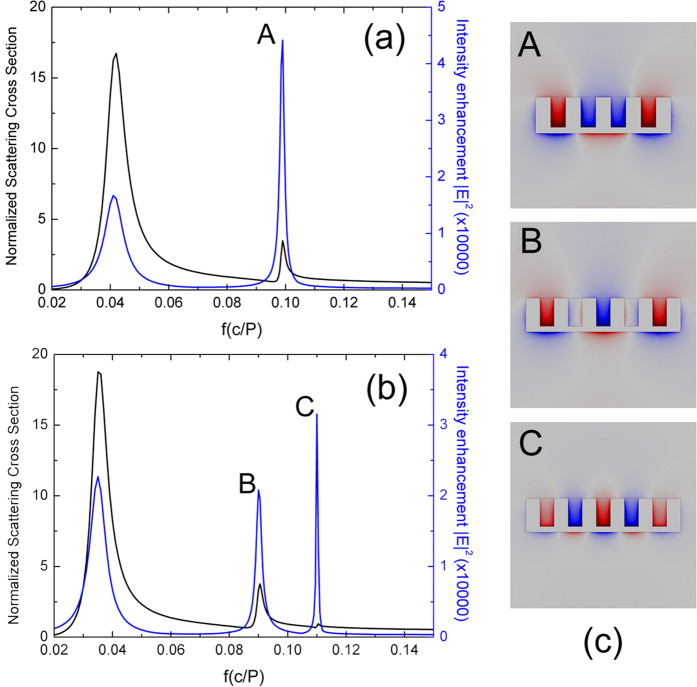
(**a**,**b**) Normalized SCS and field enhancement with different number of groove: (**a**) N = 4; (**b**) N = 5. The right panels show the magnetic field distribution (H_z_) at the marked resonances for two stripe antennas N = 4 and N = 5.

**Figure 4:**
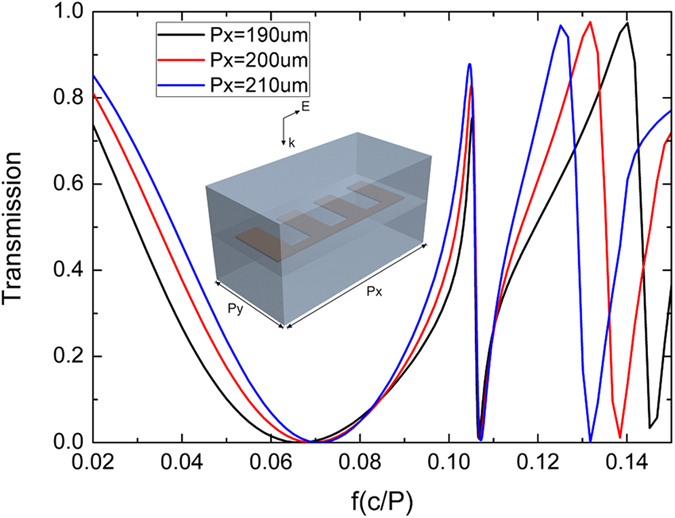
Transmission spectrum of periodic antenna in two dimensional with N = 3. Inset: Schematic diagram of periodic antenna unit cell at normal incidence.
